# Periodontal Therapy for Improving Lipid Profiles in Patients with Type 2 Diabetes Mellitus: A Systematic Review and Meta-Analysis

**DOI:** 10.3390/ijms20153826

**Published:** 2019-08-05

**Authors:** Siddharth Garde, Rahena Akhter, Mai Anh Nguyen, Clara K. Chow, Joerg Eberhard

**Affiliations:** 1 The University of Sydney School of Dentistry, Faculty of Medicine and Health, The University of Sydney, Camperdown 2006, Australia; 2 Westmead Applied Research Centre, Sydney Medical School, Westmead 2145, Australia

**Keywords:** periodontal therapy, type 2 diabetes mellitus, lipid profiles, inflammation

## Abstract

Periodontitis is a chronic inflammatory disorder often seen in patients with diabetes mellitus (DM). Individuals with diabetes are at a greater risk of developing cardiovascular complications and this may be related, in part, to lipid abnormalities observed in these individuals. The objective of this systematic review is to compile the current scientific evidence of the effects of periodontal treatment on lipid profiles in patients with type 2 diabetes mellitus. Through a systematic search using MEDLINE, EMBASE, PubMed, and Web of Science, 313 articles were identified. Of these, seven clinical trials which met all inclusion criteria were chosen for analysis. Between baseline and 3-month follow-up, there was a statistically significant reduction in the levels of total cholesterol (mean differences (MD) −0.47 mmol/L (95% confidence interval (CI), −0.75, −0.18, *p* = 0.001)), triglycerides (MD −0.20 mmol/L (95% CI −0.24, −0.16, *p* < 0.00001)) favouring the intervention arm, and a statistically significant reduction in levels of high density lipoprotein (HDL) (MD 0.06 mmol/L (95% CI 0.03, 0.08, *p* < 0.00001)) favouring the control arm. No significant differences were observed between baseline and 6-month follow-up levels for any lipid analysed. The heterogeneity between studies was high. This review foreshadows a potential benefit of periodontal therapy for lipid profiles in patients suffering from type 2 DM, however, well designed clinical trials using lipid profiles as primary outcome measures are warranted.

## 1. Introduction

Periodontal diseases are a group of inflammatory conditions affecting the connective tissues surrounding teeth. Periodontitis, a specific type of periodontal disease, is a major cause of tooth loss and the prevalence of its moderate to severe forms in adult Western populations is approximately 50% [1,2]. Periodontitis is caused by gram-negative bacteria which induce a host inflammatory response, resulting in the destruction of tissues that supports the teeth and also has adverse systemic effects [3].

Type 2 diabetes mellitus (type 2 DM) is a metabolic disorder ranging from insulin resistance to insulin deficiency, with poor glycaemic control presenting as a predominant feature [3]. Diabetes is also a major risk factor for periodontitis, and the risk of developing periodontitis is increased approximately three times in patients with diabetes compared with non-diabetic individuals [4]. There is an increasing prevalence of type 2 DM worldwide, and this is expected to contribute to an increase in diabetes-related complications [5].

Cardiovascular disease (CVD) is also one of the major complications associated with diabetes, and there is a high prevalence of cardiovascular risk factors and markers of cardiovascular organ injury in patients with type 2 DM. Ninety-seven percent of patients with diabetes are dyslipidaemic, with a characteristic pattern of increased plasma triglycerides and decreased high density lipoprotein (HDL) cholesterol. In a large clinical study with an average follow-up period of 3.9 years, low density lipoprotein (LDL) cholesterol, non-HDL cholesterol, apolipoprotein B, triglyceride, and homocysteine levels all increased over time, with most participants also having low HDL levels [6]. The downregulation of the enzyme lipoprotein lipase due to low insulin levels may be the cause of the dyslipidaemic profiles noted in diabetic individuals [7]. Other mechanisms involved linking diabetes to higher CVD risk involve chronic oxidative stress in diabetics, purportedly related to the metabolism of excess substrates (glucose and fatty acids [8]) and a state of chronic, low-level inflammation [9] in diabetes.

Recent intervention trials have demonstrated that anti-inflammatory periodontitis therapy may reduce serum levels of glycated haemoglobin (HbA1c) and high sensitivity C-reactive protein (hsCRP) [10,11,12,13,14,15,16], demonstrating the capacity to modulate glucose control and cardiovascular risk. However, little attention has been paid to the potential effects of periodontitis therapy in patients with diabetes to improve lipid profiles. This systematic review aims to evaluate the scientific evidence of the impact of periodontal therapy on lipid profiles in patients with type 2 DM.

## 2. Results

### 2.1. Selection of Studies

**Figure 1:**
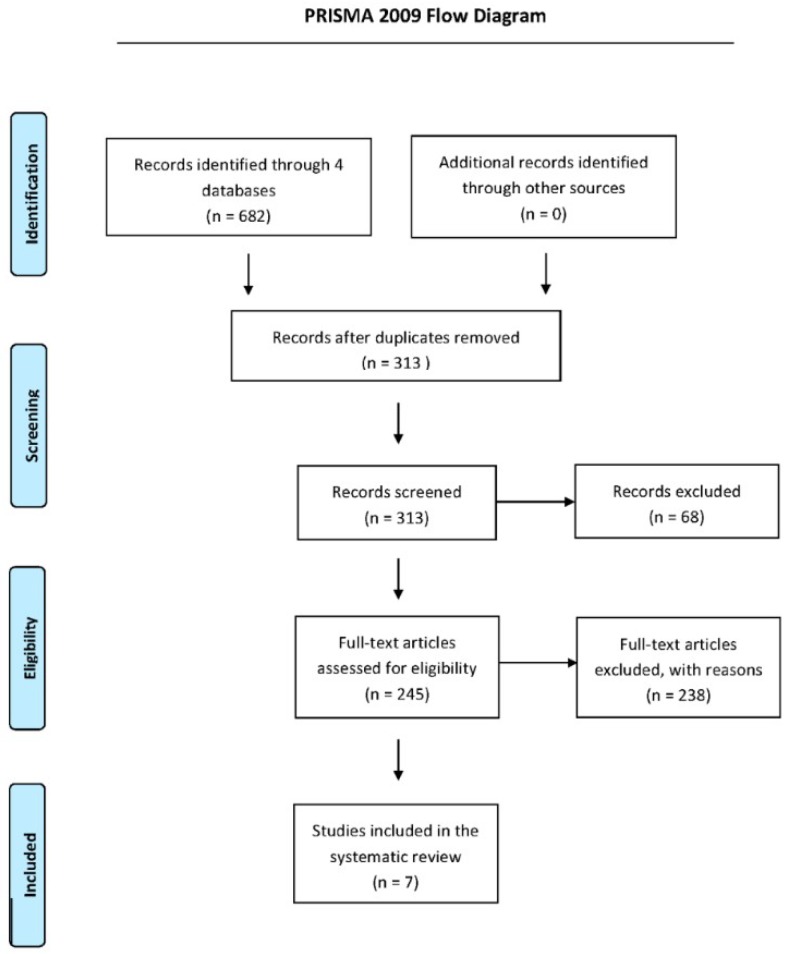
Study selection flow diagram.

Six hundred and eighty-two studies were retrieved from the electronic databases PubMed, MEDLINE via Ovid, EMBASE via Ovid, and Web of Science. Three hundred and sixty-nine duplicates were removed, and the remaining abstracts were screened for eligibility, resulting in sixty-eight studies being further excluded. Two hundred and forty-five studies were then selected for full-text analysis. After reading of the full texts, seven studies [10,11,12,13,14,15,16] were included in the systematic review (Figure 1). Full text studies were excluded if they did not report lipid levels as an outcome or studies did not have appropriate intervention and control arms.

### 2.2. Characteristics of Studies

The characteristics of the included studies are described in Table 1. In total, there were 411 individuals who underwent periodontal treatment (intervention arm) and 341 individuals who did not or received post-trial intervention (control arm). The total number of participants in each study ranged from 40 to 264. The follow-up times for the studies ranged from 3 to 12 months, however, for this review only the 3- and 6-month follow-up data has been analysed. Among seven studies, five studies reported 3-month follow-up [10,12,13,15,16] and three studies reported 6-month follow-up data [10,11,14].

The mean age of individuals in the studies ranged from 45.5 to 63.2 years old. Three out of seven studies [11,12,16] excluded patients with cardiovascular disease. Three studies [10,14,15] excluded patients with uncontrolled systemic diseases, however, did not specify whether cardiovascular disease was amongst this exclusion criteria. One study [13] did not specify whether participants with any uncontrolled systemic diseases were excluded or not. Two studies [11,14] specified that participants taking anti-hypertensive/cholesterol medications were included whereas five studies [10,12,13,15,16] did not specify whether patients taking anti-hypertensive/cholesterol medications were included or not. Six out of the seven studies [10,11,12,14,15,16] presented periodontal inclusion criteria and, among them, four different criteria were identified. All studies [10,11,12,13,14,15,16] reported diabetes inclusion criteria, and seven different criteria were identified (Table 1).

### 2.3. Risk of Bias within Studies

**Figure 2:**
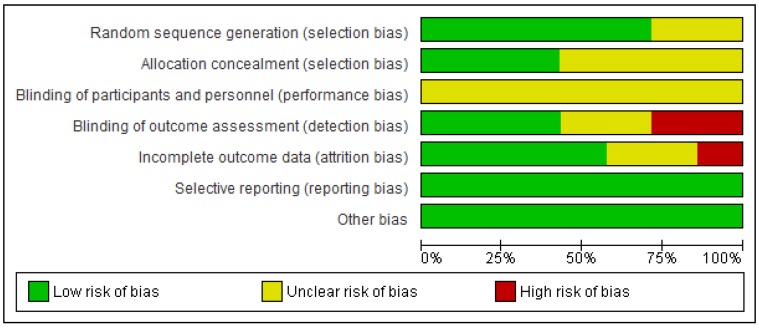
Risk of bias analysis of individual studies.

The Cochrane Collaboration’s tool for assessing risk of bias [17] was used to assess the risk of bias within the included studies which have been summarized in Figure 2. Of the seven clinical trials included [10,11,12,13,14,15,16], five studies described the methods of randomisation [10,11,12,14,15]. Three studies [11,12,14] used a computer-generated table for allocation concealment, two studies [10,15] assigned an independent individual to allocate participants, and two studies [13,16] did not specify allocation concealment. Blinding of patients and personnel was not possible due to the nature of the periodontal treatment. Blinding of investigators was conducted in four studies [10,11,12,14], two did not specify whether investigators were blinded [13,15], and one study was non-blinded [16]. Four studies had minimal/no participant drop out or the data for the participants who dropped out was excluded [13,14,15,16], two studies had a moderate number of participants drop out [10,11] and one study [12] had a high participant dropout rate indicating attrition bias. There was no evidence of selective reporting or any other form of bias in any of the studies.

### 2.4. Lipid Profiles

Changes in lipid profiles between baseline and the 3- and 6-month follow-ups have been depicted in Table 2.

#### 2.4.1. Baseline vs. 3-Month Follow-Up

**Figure 3:**
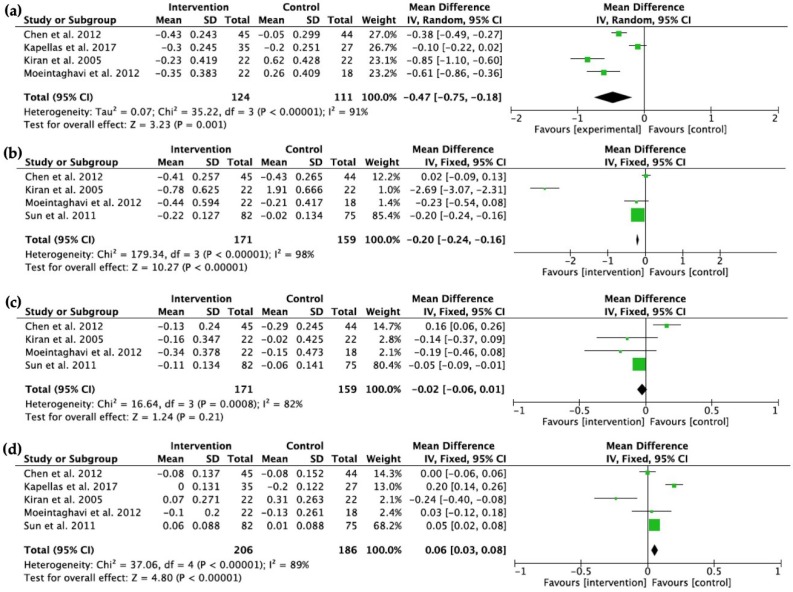
Forest plots depicting the changes of (**a**) total cholesterol, (**b**) triglycerides, (**c**) LDL, and (**d**) HDL (all in mmol/L) between the intervention and control groups at baseline and the 3-month follow-up.

After a 3 month observation period data from five studies [10,12,13,15,16] were included in the meta-analysis (Figure 3). A total of 235 participants in four studies [10,12,13,15] were analysed for changes in their total cholesterol levels and there was a statistically significant differences in favour of the intervention group (*p* = 0.001, mean difference −0.47 mmol/L (95% CI, −0.75 mmol/L to −0.18 mmol/L)) with evidence of high heterogeneity (Chi^2^ = 35.22, 3 df, *p* < 0.00001, I^2^ = 91%). A total of 330 patients in four studies [10,13,15,16] were analysed for changes in triglycerides, and there was a statistically significant difference in favour of the intervention treatment (*p* < 0.00001, mean difference −0.20 mmol/L (95% CI −0.24 mmol/L to −0.16 mmol/L, Chi^2^ = 179.34, 3 df, *p* < 0.00001, I^2^ = 98%)), with evidence of high heterogeneity. A total of 330 patients in four studies [10,13,15,16] compared changes in LDL levels, and there was no significant difference between the intervention and the control treatment (*p* = 0.21, mean difference −0.02 mmol/L (95% CI −0.06 mmol/L to 0.01 mmol/L, Chi^2^ = 16.64, 3 df, *p* = 0.0008, I^2^ = 82%)). A total of 392 patients in five studies [10,12,13,15,16] compared changes in HDL levels, and there was a statistically significant difference in favour of the control treatment (*p* < 0.00001, mean difference 0.06 mmol/L (95% CI 0.03 mmol/L to 0.08 mmol/L, Chi^2^ = 37.06, 4 df, *p* < 0.00001, I^2^ = 89%)), with evidence of high heterogeneity.

#### 2.4.2. Baseline vs. 6-Month Follow-Up

**Figure 4:**
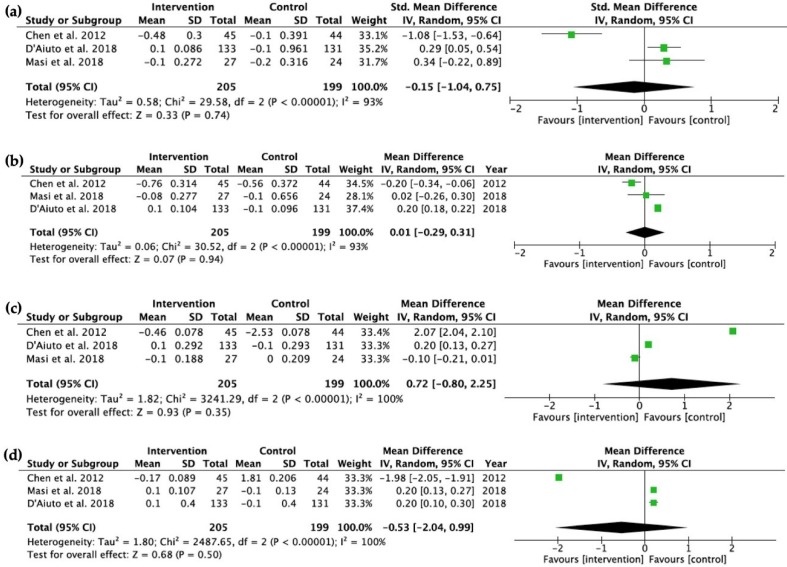
Forest plots depicting the changes of (**a**) total cholesterol, (**b**) triglycerides, (**c**) LDL, and (**d**) HDL (all in mmol/L) between the intervention and control groups at baseline and the 6-month follow-up.

After 6 months, three studies [10,11,14] were included in a meta-analysis of total cholesterol, triglycerides, LDL and HDL. There were no statistically significant differences found for any lipid levels (Figure 4).

### 2.5. Periodontal Outcomes

The periodontal parameters at baseline, 3 months, and 6 months are outlined in Table 3. At baseline, four studies [10,13,15,16] reported a mean periodontal probing depth of 2.93 mm for the intervention groups, compared to 2.82 mm in the control groups. Three months after therapy a mean PD of 2.30 mm was reported in the intervention groups (PD reduction of 0.63 mm), compared to 2.81 mm in the control groups (PD reduction of 0.1 mm). For the comparison between baseline vs. 6 months, three studies [10,11,14] reported a mean baseline PD of 3.46 mm in the intervention group and of 3.99 mm in the control group. After 6 months, a mean PD of 2.63 mm in the intervention group (PD reduction 0.83 mm) and a mean PD of 3.14 mm in the control groups (PD reduction 0.18 mm) was reported.

Two studies [10,13] reported changes in bleeding on probing (BOP) between baseline and 3-month follow-up. For the intervention group a mean BOP of 43.4% was calculated at baseline and of 18.0% at 3-month follow-up. For the control group the mean BOP at baseline was 42.2%, compared to 40.2% at 3-month follow-up. Three studies [10,11,14] reported BOP values for baseline and 6-month follow-up. For the intervention group a mean BOP of 56.5% was calculated at baseline and of 27.0% at the 6-month follow-up assessment. For the control group, the mean BOP at baseline was 56.3%, compared to 47.8% at 6-month follow-up. 

## 3. Discussion

This is the first meta-analysis aimed to evaluate the effect of anti-inflammatory periodontal therapy on changes of lipid levels in patients with type 2 DM. Periodontal therapy involves the mechanical removal of dental plaque associated with periodontitis. The majority of the studies used non-surgical periodontal treatment as the intervention, however one study [11] used surgical periodontal treatment for select individuals in the intervention arm as well. The analyses demonstrated that total cholesterol and triglycerides were significantly reduced in the intervention arm 3 months after therapy to lower levels, while HDL levels were reduced in the control group. However, no significant differences were observed after 6 months. The studies included in this review showed considerable heterogeneity, which has to be recognized before any conclusion can be drawn. However, this systematic review highlighted the potential benefits of periodontitis therapy to reduce total cholesterol and triglycerides levels. These positive effects may reduce the risk for cardiovascular complications in patients with type 2 DM.

The studies included were selected using stringent selection criteria described in the methods section, however, none of the studies included were designed to analyse lipid profiles as primary outcome measures. This may contribute to the high heterogeneity of the outcomes, as well as factors affecting lipid levels in general, including how long the individuals have had type 2 DM, lifestyle factors, and diet, which have not been assessed or reported in the included studies. All studies demonstrated a substantial reduction of clinical parameters of periodontal disease, including PD and BOP in the intervention groups 3 and 6 months after therapy, indicative of a successful treatment of the inflammatory reaction involved in periodontal disease. By contrast, the control groups did not show obvious changes in assessed oral health parameters. The mean levels of investigated total cholesterol and triglycerides decreased 3 months after periodontitis therapy, however, no differences were observed after 6 months. A possible explanation for this fading effect on lipid profiles after prolonged observation periods is recolonization by the subgingival microbiota and subsequent inflammation [18] if supportive periodontal treatment is not provided. Even though the periodontal parameters were significantly improved at the 6-month follow-up relative to baseline, five out of the seven included studies [10,12,13,15,16] did not provide supportive periodontal therapy to participants in the intervention arm after the initial treatment. In these participants, it is very likely that the recolonization of the microflora re-induced the inflammatory reaction which may have adversely affected lipid parameters. It should also be noted that average periodontal probing depths and bleeding on probing percentages are lower at the 3-month follow-up compared to the 6-month follow-up. This observation indicates the necessity of a regular periodontal maintenance program aimed to minimise the recolonization of tooth surfaces with periodontal pathogens and the concordant inflammation of the adjacent tissues.

Three out of the seven studies included in the current review [14,15,16] showed a significant reduction in levels of glycated haemoglobin and four studies [10,11,12,13] did not show significant changes between baseline and follow-up. In those studies reporting a significant reduction of glycated haemoglobin after periodontal treatment, one study [15] showed improved levels of total cholesterol, one study [16] showed an improvement in levels of HDL, whereas in both studies, other lipid parameters showed no significant changes. The third study [16] did not find any difference in lipid parameters between baseline and follow-up despite the reduction in levels of glycated haemoglobin. Within the limitations of this comparison, a reduction in glycated haemoglobin may not necessarily be accompanied by changes in lipid levels.

Individuals with periodontitis have been noted to have an increased risk of hyperlipidaemia and hypercholesterolaemia [19]. As mentioned previously, periodontitis is a chronic infection of the tooth supporting structures [1], and local chronic infections have been shown to alter concentrations of cytokines and hormones which can result in changes in lipid metabolism [20]. Specifically, systemic exposure to infectious challenges such as bacterial lipopolysaccharide can result in the release of inflammatory cytokines including interleukin-1 (IL-1) and tumour necrosis factor alpha (TNF-α) that alter fat metabolism and promote hyperlipidaemia. Both TNF-α and IL-1 inhibit the production of lipoprotein lipase, which causes disturbances of lipid metabolism, including increased amounts of serum cholesterol and LDL [21]. A second mechanism by which bacterial lipopolysaccharides contribute to the development of atherosclerosis is by oxidative modification of increased LDL caused by macrophage activation. Oxidized LDL is taken up by macrophage scavengers, which leads to transformation of macrophages into foam cells, the hallmark of the atherosclerotic process. Oxidized LDL is also cytotoxic to endothelial cells and a potent chemoattractant for circulating human monocytes [22]. Conversely, it has also been demonstrated that a short-term high-fat diet results in prolonged impairment in the antibacterial function of polymorphonuclear leukocytes, which may cause damage of periodontal tissues [23]. Thus, a chronic hyperlipidaemic state may impair the host resistance to bacterial infection.

Cardiovascular disease is a major complication of type 2 DM and lipid abnormalities seen in diabetics are a serious contributor to this complication [6]. Glycaemic control via maintaining adequate levels of HbA1c is considered as an essential way to lower patients’ risk of having diabetic complications and each 1% drop in HbA1c levels is associated with a risk reduction of 21% for diabetes-related deaths, 14% for myocardial infarction, and 37% for microvascular complications [24]. Several studies have indicated that periodontal infection caused by gram-negative bacteria had adverse effects on diabetic patients’ glycaemic control [25,26]. By contrast, improved periodontal conditions following periodontal treatment can significantly improve HbA1c levels [11,27]. A lipid-lowering management in type 2 DM patients is also aimed at reducing the incidence of cardiovascular complications, and statins can be very effective in improving the lipid profile and are therefore the first line class of drugs [28]. In general, different statins have varying abilities to improve lipid profiles in patients, e.g., HDL cholesterol levels increase between 5% and 10% with statin therapy, LDL levels reduce, ranging from 27% to 60%, and triglycerides levels reduce between 11% and 40% [29]. The current analysis demonstrated a mean reduction of triglyceride levels by approximately 8% achieved by periodontitis treatment. Within the limitations of the available study data and the heterogeneity of studies, this will not be sufficient to annotate periodontitis treatment as an adjunct to a lipid-lowering management in patients suffering from type 2 DM. However, it may stimulate the interest in further exploring the benefits of good oral health for the prevention of diabetes complications and especially to setup well designed clinical trials with lipid profiles as the primary outcome.

## 4. Materials and Methods

### 4.1. Types of Studies

Randomized control trials of 3- or 6-month follow-ups were considered for this review.

### 4.2. Types of Participants

The participants of the included studies had a diagnosis of type 2 DM and periodontitis. Patients with type I diabetes were excluded.

### 4.3. Types of Intervention

All periodontal treatments using mechanical debridement (surgical and non-surgical, with and without adjunctive treatment) were included.

### 4.4. Types of Outcome Measures

Primary outcome measures were total cholesterol, triglycerides, LDL cholesterol, and HDL cholesterol between baseline and 3- or 6-month follow-ups. Secondary outcome measures were periodontal probing depths, clinical attachment loss, and bleeding on probing.

### 4.5. Search Methods

The search attempted to identify all relevant trials in English. The electronic databases searched were (date of most recent search 19 May 2019) PubMed, MEDLINE via Ovid, EMBASE via Ovid and Web of Science. A sensitive search strategy was developed following the PICO process for the question: Does periodontal treatment improve lipid profiles in individuals with type 2 DM?

- Patients = individuals with type 2 DM
- Intervention = anti-inflammatory surgical or non-surgical periodontal treatment
- Comparison = no periodontal treatment or only supragingival scaling and polishing
- Outcome = lipid profiles

The search strategy for PubMed is given as an example: (“periodontal treatment” OR “periodontitis treatment” OR “periodontal therapy” OR “periodontitis therapy” AND “diabet*”). Incomplete information and ambiguous data were researched further by contacting the author and/or researcher responsible for the study directly. If the corresponding author failed to reply, the studies were excluded. Cross-sectional studies, retrospective studies, literature reviews, systematic reviews, editors’/authors’/reviewers’ comments, articles not in English, studies where the intervention was not periodontal treatment, studies which did not have an appropriate control arm, studies where lipids were not analysed both pre and post-trial, and trials involving individuals with diabetes other than type 2 DM were excluded.

### 4.6. Selection of Studies

Titles and abstracts were managed by downloading to Endnote X8 software. The selection of papers, the decision about eligibility, and data extraction were carried out independently, in duplicate, by three reviewers (S.G., J.E. and M.A.N.). Any disagreement was resolved by discussion. The full text of the included studies was evaluated by two authors (S.G. and M.A.N.). Data entry to a computer and data extraction was carried out by one reviewer (S.G.).

### 4.7. Data Extraction

The following data was extracted:

- General study characteristics: authors, year of study, country of origin, intervention/control, number of participants at baseline, follow-up period, diabetes and periodontal inclusion criteria
- Primary outcomes: lipid profiles (total cholesterol, triglycerides, LDL, HDL)
- Secondary outcomes: probing depth and bleeding on probing at baseline and 3- or 6-month follow-ups.

### 4.8. Quality Assessment

Quality assessment was done according to the guidelines of the Cochrane Handbook for Systematic Reviews of Interventions [17].

### 4.9. Data Synthesis

For continuous outcomes, mean differences (MD) and 95% CI were used to summarize the data for each group. All statistical analyses were conducted with Review Manager 5.3. Heterogeneity was assessed with Cochran’s test for heterogeneity undertaken prior to each meta-analysis, and I^2^ statistics.

## Figures and Tables

**Table 1:** Characteristics of included studies.

Author	Country	Intervention/Control	Participants at Baseline (*n*)	Follow Up TIME (months)	Diabetes Inclusion Criteria	Periodontal Inclusion Criteria
D’Aiuto et al. 2018 [11]	United Kingdom	SPT + NSPT	133	12	Type 2 DM for >6 months (WHO diagnostic criteria)	>20 periodontal pockets with PD > 4mm and alveolar bone loss > 30%
		Supragingival SRP	131			
Masi et al. 2018 [14]	United Kingdom	NSPT	27	6	Diagnosed type 2 DM (WHO criteria)	>15 remaining teeth and >20 sites with PD >5mm
		Supragingival SRP	24			
Kapellas et al. 2017 [12]	Australia	NSPT	35	3	HbA1c > 6.5% or >47.5 mmol/mol	Joint Centers for Disease Control and Prevention and American Academy of Periodontology case definition
		No treatment	27			
Chen et al. 2012 [10]	China	NSPT	45	6	Type 2 DM for >12 months	American Academy of Periodontology criteria
		No treatment	44			
Moeintaghavi et al. 2012 [15]	Iran	NSPT	22	3	HbA1c > 7%	Mild-moderate periodontitis- American Academy of Periodontology criteria
		No treatment	18			
Sun et al. 2011 [16]	China	NSPT	82	3	Diagnosed type 2 DM for >12 months and HbA1c 7.5–9.5%	>20 remaining teeth, PD > 5 mm, more than 30% teeth CAL over 4 mm, or over 60% teeth with PD > 4 mm and CAL > 3 mm
		No treatment	75			
Kiran et al. 2005 [13]	Turkey	NSPT	22	3	HbA1c 6–8%	Not specified
		No treatment	22			

SPT: surgical periodontal treatment; NSPT: non-surgical periodontal treatment; SRP: scaling and root planing; DM: diabetes mellitus; HbA1c: glycated haemoglobin; WHO: World Health Organization; PD: pocket depth; CAL: clinical attachment loss.

**Table 2:** Lipid profiles at baseline, 3 months, and 6 months in mmol/L.

Author	Groups		Baseline								3 Month Follow Up								6 Month Follow Up							
			TC		TG		LDL		HDL		TC		TG		LDL		HDL		TC		TG		LDL		HDL	
			Mean	SD	Mean	SD	Mean	SD	Mean	SD	Mean	SD	Mean	SD	Mean	SD	Mean	SD	Mean	SD	Mean	SD	Mean	SD	Mean	SD
D’Aiuto et al. 2018 [11]	Intervention	SPT+NSPT	4.2	1 *	1.6	1.2 *	2.2	0.9 *	1.2	0.4 *									4.3	0.1 *	1.7	0.1 *	2.3	0.1 *	1.3	0.0 *
	Control	SG SRP	4.3	1.1 *	1.6	1.1 *	2.4	0.9 *	1.3	0.4 *									4.2	0.1 *	1.5	0.1 *	2.3	0.1 *	1.2	0.0 *
Masi et al. 2018 [14]	Intervention	NSPT	4.3	1.1	1.48	1.13	2.3	0.9	1.3	0.4									4.2	0.9	1.4	0.9	2.2	0.7	1.4	0.4
	Control	SG SRP	4.3	1	2.3	2.6	2	0.9	1.3	0.4									4.1	1.1	2.2	1.9	2	0.8	1.2	0.5
Kapellas et al. 2017 [12]	Intervention	NSPT	4.8	1.1					1	0.3	4.5	1					1	0.3								
	Control	No tx	4.6	0.8					1.1	0.2	4.4	0.9					0.9	0.2								
Chen et al. 2012 [10]	Intervention	NSPT	2.63	1.32	6.02	1.57	3.5	1.3	1.31	0.46	2.2	1.33	5.61	1.41	3.37	1.3	1.23	0.39	2.15	1.93	5.26	1.41	3.04	1.23	1.14	0.39
	Control	No tx	2.35	1.78	6.37	1.87	3.79	1.48	1.44	0.53	2.3	2.16	5.94	1.22	3.5	1.17	1.36	0.49	2.25	1.98	5.81	1.61	1.26	0.5	3.25	1.27
Moeintaghavi et al. 2012 [15]	Intervention	NSPT	10.66	1.5	7.66	4.54	6.6	1.38	2.55	0.51	10.31	1.72	7.22	3.22	6.26	1.76	2.45	0.37								
	Control	No tx	10.69	1.5	8.4	1.51	6.51	1.9	2.57	0.69	10.95	1.51	8.19	1.62	6.36	2.12	2.44	0.54								
Sun et al. 2011 [16]	Intervention	NSPT			2.07	0.69	3.32	0.71	1.17	0.29			1.85	0.64	3.21	0.76	1.23	0.33								
	Control	No tx			2.1	0.68	3.37	0.74	1.15	0.28			2.08	0.66	3.31	0.75	1.16	0.3								
Kiran et al. 2005 [13]	Intervention	NSPT	10.4	2.13	7.6	5.5	6.31	1.35	2.87	0.77	10.17	1.73	6.82	3.1	6.15	1.73	2.94	0.85								
	Control	No tx	9.95	1.95	7.26	3.8	5.96	1.81	2.54	0.73	10.57	2.07	9.17	5.95	5.94	2.16	2.85	0.79								

TC: total cholesterol; TG: triglyceride; LDL: low-density lipoprotein; HDL: high-density lipoprotein; SD: standard deviation; *: standard error; NSPT: non-surgical periodontal treatment; SG SRP: supragingival scaling and root planning; tx: treatment.

**Table 3:** Periodontal parameters at baseline, and 3 and 6 months.

Author	Groups		Baseline				3-month Follow Up				6-month Follow Up			
			PD		BOP		PD		BOP		PD		BOP	
			Mean	SD	Mean	SD	Mean	SD	Mean	SD	Mean	SD	Mean	SD
D’Aiuto et al. 2018 [11]	Intervention	SPT + NSPT	3.9	0.1 *	65	2.0 *					2.9	0.1 *	33.0	2.0 *
	Control	SG SRP	3.9	0.1 *	63	2.0 *					3.7	0.1 *	57.0	2.0 *
Masi et al. 2018 [14]	Intervention	NSPT	3.9	0.8	70.0	20.0								
	Control	SG SRP	3.6	0.7	72.0	15.0								
Kapellas et al. 2017 [12]	Intervention	NSPT												
	Control	No treatment												
Chen et al. 2012 [10]	Intervention	NSPT	2.57	0.66	32.42	16.63	2.2	0.39	12.13	8.24	2.1	0.39	12.02	8.99
	Control	No treatment	2.47	0.57	34.01	18.91	2.38	0.47	28.53	14.42	2.42	0.5	28.37	13.5
Moeintaghavi et al. 2012 [15]	Intervention	NSPT	2.31	0.65			2.21	0.6						
	Control	No treatment	2.06	0.24			2.33	0.3						
Sun et al. 2011 [16]	Intervention	NSPT	4.53	0.83			2.97	0.78						
	Control	No treatment	4.49	0.85			4.28	0.81						
Kiran et al. 2005 [13]	Intervention	NSPT	2.29	0.49	54.38	18.75	1.8	0.25	23.9	12.73				
	Control	No treatment	2.24	0.7	50.48	26.1	2.26	0.63	51.91	27.38				
**Total**	**Intervention**		**3.3**	**0.6**	**55.5**	**16.1**	**2.3**	**0.5**	**18.0**	**10.7**	**2.6**	**0.5**	**27.0**	**13.1**
	**Control**		**3.1**	**0.6**	**54.9**	**17.8**	**2.8**	**0.3**	**40.2**	**21.9**	**3.1**	**0.5**	**47.8**	**13.0**

SPT: surgical periodontal treatment; NSPT: non-surgical periodontal treatment; SG SRP: supragingival scaling and root planing; PD: pocket depth (mm); SD: standard deviation; *: standard error; BOP: bleeding on probing (%).
